# Acidic pH triggers the phosphorylation of the response regulator NtrX in alphaproteobacteria

**DOI:** 10.1371/journal.pone.0194486

**Published:** 2018-04-10

**Authors:** Ignacio Fernández, Gabriela Sycz, Fernando Alberto Goldbaum, Mariela del Carmen Carrica

**Affiliations:** Fundación Instituto Leloir, IIBBA-CONICET, Patricias Argentinas, Buenos Aires, Argentina; East Carolina University Brody School of Medicine, UNITED STATES

## Abstract

Many signaling pathways that control cellular development, cell-cycle progression and nutritional versatility have been studied in *Caulobacter crescentus*. For example, it was suggested that the response regulator NtrX is conditionally essential for this bacterium and that it might be necessary for responding to a signal produced in phosphate-replete minimal medium. However, such signal has not been identified yet and the role of NtrX in *C*. *crescentus* biology remains elusive. Here, using wild-type *C*. *crescentus* and a strain with a chromosomally myc-tagged *ntrX* gene, we demonstrate that high concentrations of phosphate (10 mM) regulate *ntrX* transcription and the abundance of the protein. We also show that the pH of the medium acts as a switch able to regulate the phosphorylation status of NtrX, promoting its phosphorylation under mildly acidic conditions and its dephosphorylation at neutral pH. Moreover, we demonstrate that the *ntrX* gene is required for survival in environments with low pH and under acidic stress. Finally, we prove that NtrX phosphorylation is also triggered by low pH in *Brucella abortus*, a pathogenic alphaproteobacterium. Overall, our work contributes to deepen the general knowledge of this system and provides tools to elucidate the NtrX regulon.

## Introduction

Perception of environmental and intracellular cues is an essential feature of life. Signaling pathways enable cells to regulate genetic and biochemical programs for adaptation and survival. Among the most important strategies that bacteria employ for performing those tasks are the two-component systems (TCSs). They comprise a histidine kinase (HK) that autophosphorylates upon perception of a stimulus and then transfers the phosphoryl group to a cognate response regulator (RR) [1]. The phosphorylated RR is activated to perform output functions such as modulation of gene expression, interaction with partner proteins, etc. [1].

*Caulobacter crescentus* is a gram-negative bacterium that grows in dilute aquatic environments and is a member of the alpha-subdivision of proteobacteria. Much attention has been given to the study of *C*. *crescentus* signaling pathways to describe how they control cellular development and cell-cycle progression [2], and also to understand how this oligothopic bacterium is able to display nutritional versatility and to adapt to nutrient-poor environments. For example, a system-level investigation of TCSs showed that at least 39 of the 106 two-component genes are required for cell cycle progression, growth, or morphogenesis [3]. Among them, the gene that codes for the RR NtrX (CC_1743) was considered conditionally essential because a mutant strain with the deleted gene could not be obtained in rich medium (PYE) but the deletion procedure performed on minimal medium (M2G) yielded a stable deletion strain [3]. Further characterization of this mutant indicated that it has a growth deficiency and fitness disadvantage in phosphate-replete minimal medium (M2G), but this difference with respect to the wild-type strain is not manifested in phosphate-limited minimal medium (M5G) [4]. Although these observations suggested that NtrX might be necessary for responding to a signal or metabolite present in the M2G medium, such signal was not identified and the role of NtrX in *C*. *crescentus* biology remains elusive.

NtrX forms a TCS with its cognate HK NtrY, which is predicted to be a membrane protein with a periplasmic domain and intracellular HAMP, PAS and HK domains. The NtrY/X pathway has been extensively studied in the pathogen *Brucella abortus*, in which it has been reported that it participates in sensing low oxygen tension and in the regulation of the expression of denitrification enzymes and high-oxygen affinity cytochrome oxidases [5,6]. This TCS is also present in other microorganisms where it has been involved in a variety of functions that includes: nitrogen fixation and metabolism in *Azorhizobium caulinodans* [7], *Rhodobacter capsulatus* [8] and *Herbaspirillum seropedicae* [9]; regulation of proline and glutamine metabolism in *Ehrlichia chaffeensis* [10]; expression of respiratory enzymes in *Neisseria gonorrhoeae* [11]; and succinoglycan production, motility, and symbiotic nodulation in *Sinorhizobium meliloti* [12,13].

In this article we report that NtrX expression is induced by 10 mM phosphate and that acidic pH leads to NtrX phosphorylation. We also show that this signal is physiologically relevant since *C*. *crescentus* produces the acidification of the M2G medium upon entry into stationary phase, causing NtrX phosphorylation at this stage of the growth curve. Besides, we demonstrate that *ntrX* deletion produces a decreased viability at stationary phase and a reduced resistance to acidic stress. Finally, we prove that NtrX is also phosphorylated by acidic pH in *B*. *abortus*, pointing out to a potentially conserved role across the alphaproteobacteria class.

## Materials and methods

### Bacterial strains and culture conditions

*C*. *crescentus* cells were grown at 30°C in M2G (10 mM phosphate, glucose as carbon source), M5G (50 μM phosphate, glucose as carbon source), M2X (10 mM phosphate, xylose as carbon source) or peptone yeast extract (PYE) media [14] supplemented when necessary with nalidixic acid 10 μg/ml, tetracycline 2 μg/ml or kanamycin 5 μg/ml (liquid) or 25 μg/ml (solid). Cultures reached logarithmic phase when their OD_600_ was 0.2–0.3, while stationary phase was defined by an OD_600_ of 1.2 or higher. When required, the pH of the liquid media was adjusted using HCl, unless otherwise indicated. *C*. *crescentus* strains CB15N and Δ*ntrX* were generously donated by Laub MT, Department of Biology, Massachusetts Institute of Technology, Cambridge, MA, USA.

*B*. *abortus* cells were grown at 37°C in minimal medium [15] or tryptose agar (TA) (DIFCO), supplemented when appropriate with nalidixic acid 10 μg/ml and/or kanamycin 25 μg/ml. The cultures reached logarithmic phase at OD_600_ 0.2–0.4 and stationary phase at OD_600_ > 1.2. When appropriate, the pH of the minimal medium was adjusted to different values with HCl.

*E*. *coli* strains were grown at 37°C in LB supplemented with kanamycin (50 μg/ml).

### Construction of CC_NtrX_myc_ strain

To construct a *C*. *crescentus* strain with a chromosomally myc-tagged NtrX protein (CC_NtrX_myc_) we amplified *ntrX* from *C*. *crescentus* CB15N genomic DNA with primers PstI-ntrX_ff_ and ntrX-Myc-PstI_rev_ (5’-AACTGCAGATGAGCGCCGACGTTCTTGTG-3’ and 5’-TTCTGCAGTTACAGATCTTCTTCCGAGATCAGCTTCTGTTCCTCTTCCTCATCGCCCCGAG-3’, respectively). Then, the PCR product was digested with PstI and ligated into the pNPTS138 plasmid. The resulting vector was transformed into *E*. *coli* S17-1 and transferred to *C*. *crescentus* CB15N by conjugation. Homologous recombination led to the integration of that plasmid, resulting in *ntrX_myc* in the locus that was previously occupied by the endogenous gene (therefore, under the same transcriptional regulation) and the wild-type endogenous copy of *ntrX* coded now after the integrated pNPTS138 backbone. The integration of the plasmid was selected by kanamycin resistance and verified by PCR.

### Construction of the complemented strain CC_ΔntrX-NtrX_myc_

DNA encoding full-length tagged NtrX was amplified from pNPTS138-ntrX-myc using primers pMR10-NtrX_ff_ and Myc-pMR10_rev_, 5’-tcctgcagagctctagagtcgagacATGAGCGCCGACGTTCTTGTGGTGG-3’ and 5’-TTAAGTGCGGCCCCCTCGAGGGGGTCTACAGATCTTCTTCCGAGATCAGCTTCTGTTC-3’, respectively. The PCR product was used as a megaprimer in a PCR reaction with the pMR10 plasmid as template, according to the restriction-free cloning method [16]. Then, the PCR reaction was digested with DpnI at 37°C for 2 h and the mixture was transformed into competent *E*. *coli* DH5α cells. Selection was carried out on LB-kanamycin plates, and the resulting plasmid (pMR10-ntrXmyc) was isolated and sequenced. Finally it was transformed into *E*. *coli* S17-1 and transferred to *C*. *crescentus* Δ*ntrX* by conjugation. Complemented strains were selected by kanamycin resistance and then NtrX_myc_ expression was verified by Western blot against the tag.

The plasmid encoding the mutant protein NtrX_myc_(D53A) was obtained from pMR10-NtrX_myc_ by PCR amplification using primers 5′-GCTTTGCTGGTGCTGGCCATCTGGATGCAGG-3′ and 5′-CCTGCATCCAGATGGCCAGCACCAGCAAAGC-3′, followed by digestion with the enzyme DpnI. Further steps to obtain the *C*. *crescentus* strain were conducted as detailed in the previous paragraph.

### Construction of BA_NtrX_myc_ strain

To construct a *B*. *abortus* strain with a chromosomally myc-tagged NtrX protein (BA_NtrX_myc_) we amplified *ntrX* from *B*. *abortus* 2308 genomic DNA with primers BA-ntrX_ff_ and BA-ntrXmyc_rev_, 5’-AACTGCAGATGGCGGCCGATATTCTTGTTGTTG-3’ and 5’-TTCTGCAGTTACAGATCTTCTTCCGAGATCAGCTTCTGTTCTACGCCGAGAGACTTCAGCTTGCGA-3’, respectively. Then, the PCR product was digested with PstI and ligated into the pNPTS138 plasmid. The resulting vector was transformed into *E*. *coli* S17-1 and transferred to *B*. *abortus* 2308 by conjugation. Homologous recombination led to the integration of the plasmid, resulting in *ntrX_myc* in the locus that was previously occupied by the endogenous gene (therefore, under the same transcriptional regulation) and the wild-type endogenous copy of *ntrX* now coded after the integrated pNPTS138 backbone. The integration of the plasmid was selected by kanamycin resistance and verified by PCR.

### Isolation of total RNA from *C*. *crescentus* bacterial cell culture

*C*. *crescentus* wild type and CC_NtrX_myc_ were grown in M2G or M5G at 30°C until stationary phase (OD_600_ 1.0–1.3). After harvest, the supernatant was removed, and the pellet was resuspended in 100 μl of a solution containing 84 μl of TE buffer, 15 μl of 10% SDS and 1 μl of 10 mg/ml proteinase K. The samples were then incubated at 37°C for 1 h and 600 μl of Qiagen RLT lysis buffer was added. Total RNA was isolated following the Qiagen RNeasy Mini Bacterial protocol. DNA was subsequently removed by digestion with RQ1 RNase-free DNAse (Promega) according to the manufacturer’s instructions. RNA was quantified using a NanoDrop spectrophotometer (ND-1000, Thermo Fisher Scientific).

### Real-time quantitative RT-PCR assay

Reverse transcription was performed with SuperScritpt III First-strand synthesis kit (Invitrogen) using random decamer primers (Invitrogen). Complementary DNA (cDNA) samples were used as templates in quantitative real-time PCRs (qRT-PCRs). Primers were designed with the Primer3 program (http://www.ncbi.nlm.nih.gov/tools/primer-blast/) obtaining primers NtrX-RT_ff_ and NtrX-RT_rev_ (5’-CTGGAGGATGAAGGCTATGC-3’ and 5’-CAGATATCCAGCACCAGCAA-3’, respectively), which amplify a 101 bp region. Real-time PCRs were performed with SYBR Green in 96-well plates in an Mx3005P Stratagene instrument and analyzed with the MxPro program. The results for the target mRNA were normalized to the amount of the *C*. *crescentus* CC_0088 mRNA for which primers 5’-CGGCTCATTCTCGATCTCTT-3’ and 5’-CCTCGACAATGCTGAACTGA-3’ were used.

### Western blot analysis

To verify the expression of the NtrX_myc_ protein, the CC_NtrX_myc_ strain was grown under the conditions indicated in the figure legends. Then, the OD_600_ of the cultures was measured and volumes corresponding to the same amount of bacteria were centrifuged. The pellets were resuspended in 1X Laemmli buffer and heated 10 min at 90°C. These samples were loaded in two polyacrylamide gels and subjected to electrophoresis. One of them was stained with Coomassie Brilliant Blue (total protein stain for loading controls) while the other was transferred to a nitrocellulose filter (Millipore). Membranes were probed with monoclonal mouse anti-myc antibody (Cell Signaling Technology) at a 1:2,000 dilution, and a secondary HRP-conjugated anti-mouse antibody (Sigma) used at a 1:3,000 dilution. Blots were developed using SuperSignal^TM^ West Pico Chemiluminiscent Substrate (Thermo Scientific), following the manufacturer’s instructions. Signal intensity was measured using ImageQuant LAS4000 (GE Healthcare Life Sciences) and quantified using the ImageJ program.

#### Phosphoprotein affinity gel electrophoresis

NtrX_myc_ phosphorylation was analyzed in cultures grown and incubated as detailed in the figure legends. The samples were prepared by centrifuging equal amounts of bacteria, according to the OD_600_ of the cultures, and then the pellets were frozen until used. *C*. *crescentus* samples were resuspended in 1X Laemmli buffer and disrupted by sonication, using one pulse of 15 seconds at an output wattage of 2 (QsonicaXL– 2000 series, Misonix). *B*. *abortus* samples were disrupted using a Precellys24 homogenizer (Bertin Technologies) with 4 cycles of 3 x 30 seconds at 6,500 rpm, incubating on ice between each cycle. The homogenate was centrifuged for 2 min at 5,000 x g at 4°C to remove unbroken cells and precellys beads, and the supernatant was then centrifuged 5 min at 10,000 x g. Laemmli buffer to a final 1X concentration was added to the resulting supernatant. To avoid NtrX dephosphorylation, the samples were not heated and they were loaded after disruption in polyacrylamide gels copolymerized with 35 μM Phos-tag™ and 150 μM ZnCl_2_. Electrophoresis was performed with standard denaturing running buffer at 4°C under constant voltage (150 V). After electrophoresis, the gels were washed with EDTA 1 mM and then the proteins were transferred to a nitrocellulose membrane (Millipore) to perform Western blots as previously described. When appropriate, the bands were quantified with the ImageJ program, and the percentage of NtrX phosphorylation (NtrX~P %) was calculated as the ratio [(NtrX~P)/(NtrX_TOT_)]x100, where NtrX_TOT_ corresponds to the total intensity of the bands of phosphorylated and unphosphorylated NtrX.

### Growth curve and determination of bacterial viability

Overnight cultures of *C*. *crescentus* CB15N, the Δ*ntrX* mutant strain and the CC_Δ*ntrX*-NtrX_myc_ complemented strain were diluted to an OD_600_ of 0.005 in M2G and were incubated at 30°C with agitation (170 rpm). Samples were periodically taken to measure the OD_600_ and to determine the bacterial viability by counting the number of colony forming units (CFU) after plating 10-fold serial dilutions onto M2G plates and incubating them at 30°C for 3 days.

### Bacterial survival in response to acidic pH stress

The different *C*. *crescentus* strains were grown in M2G medium until they reached logarithmic or stationary phases, according to the OD_600_ values that were previously mentioned. At this point, they were centrifuged, resuspended in the same volume of M2G medium adjusted to pH 4.0, and incubated at 30°C with agitation for 30 minutes. Samples were taken immediately after the addition of the acidic media (time 0) and after the incubation period (time 30) to determine the number of viable bacteria by plating 10-fold serial dilutions on solid M2G plates. The experiment was performed independently three times by triplicate and the percentage of survival was calculated as the ratio between the number of viable bacteria at time 30 and the initial viable bacteria (time 0) multiplied by 100.

### Statistical analysis

Statistical analyses were performed using a two-tail Student’s t-test, or one-way or two-way ANOVA with a Bonferroni’s multiple comparison post-test using GraphPad Prism5. Data are presented as mean ± standard deviation (SD) of the mean. P-values of ≤0.05 were considered significant. Statistical significance levels were defined as follows: *p<0.05; **p<0.01; ***p<0.001.

## Results

### NtrX expression is induced by high concentrations of phosphate

**Fig 1:**
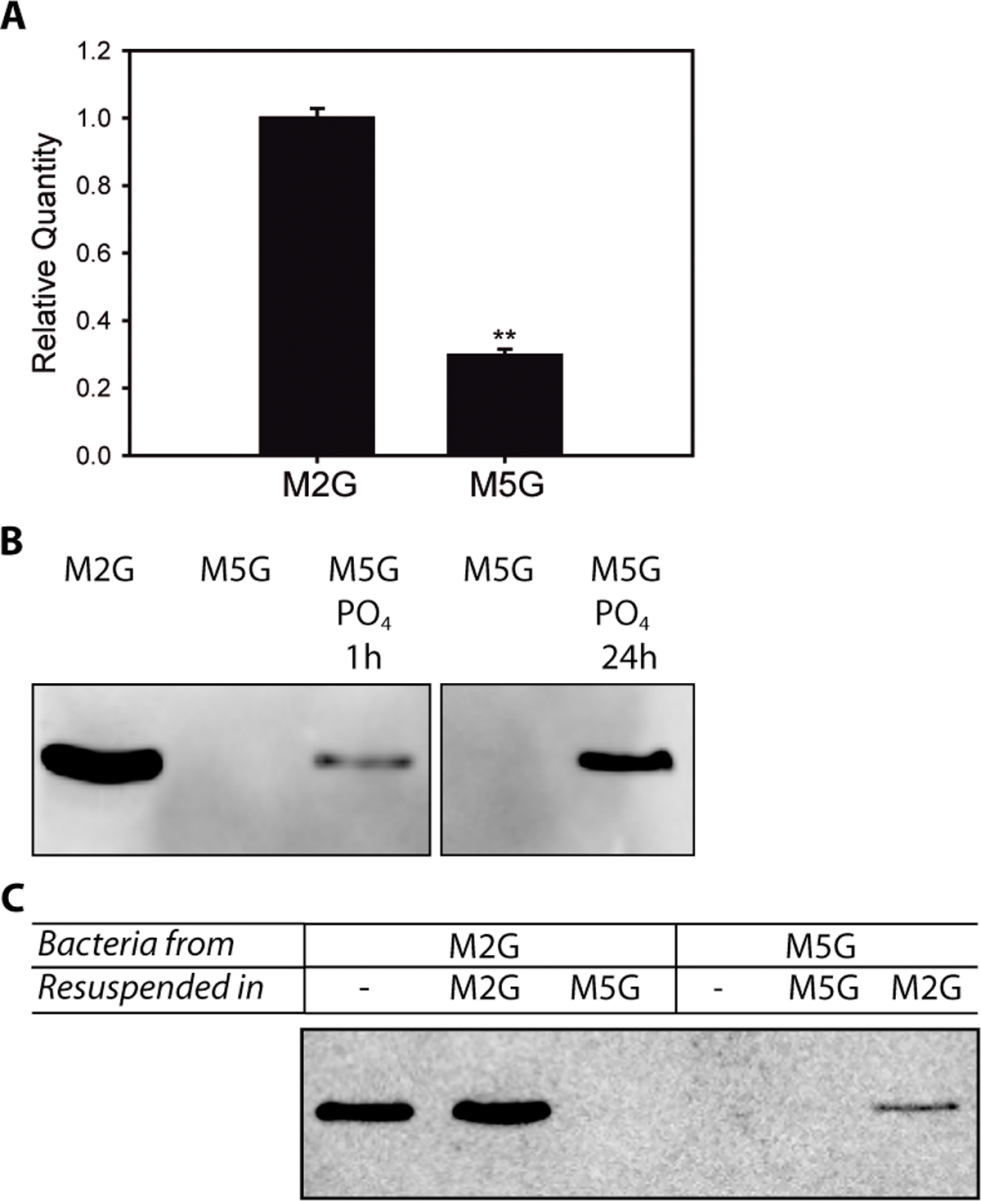
NtrX expression is induced under phosphate-replete conditions. The expression of NtrX in different media was determined by qRT-PCR and Western blot. (A) qRT-PCR to determine the level of *ntrX* transcripts in *C*. *crescentus* wild type grown in M2G and M5G until stationary phase. Data represent the mean ± standard deviation of three independent experiments, each performed by triplicate. The *p*-value was determined by a two-tailed Student’s t-test (**p<0.01). (B) The CC_NtrX_myc_ strain was grown in M2G or M5G and samples of these cultures were analyzed after 16 h. Also, at this point an aliquot of the M5G culture was taken, 10 mM sodium phosphate was added and it was further incubated for 1 h or 24 h when samples were withdrawn to be analyzed by Western blot. (C) Initial cultures of CC_NtrX_myc_ were grown overnight in M2G or M5G, and samples were taken at time zero (lane ‘-’), or they were centrifuged and resuspended in fresh M2G or M5G. After an 8 h incubation aliquots of these samples were analyzed by Western blot. Each experiment from (B) and (C) was performed independently three times, and the result of one of these repetitions is shown.

In order to understand the activation of the NtrY/X system in *C*. *crescentus*, we decided to determine under which growth conditions NtrX is expressed. Previous reports indicated that a *C*. *crescentus* Δ*ntrX* strain grows more slowly and has a fitness disadvantage in phosphate-replete minimal medium (M2G, 10 mM phosphate), but not in the phosphate-limited medium M5G (50 μM phosphate) [4]. This might suggest that the *C*. *crescentus* NtrY/X pathway is necessary for responding to a signal or metabolite produced in M2G, but it could also imply that NtrX is not present in M5G. To establish the expression of this RR, we measured the levels of the *ntrX* transcript by qRT-PCR in *C*. *crescentus* wild type (CC_WT) grown in M2G and M5G and observed that the expression of the gene is significantly lower under phosphate-limited conditions (Fig 1A).

Then, to verify if there is a correlation between the levels of the *ntrX* transcript and the abundance of the protein, we generated a strain with a chromosomally myc-tagged NtrX (CC_NtrX_myc_) that was grown in M2G and M5G. As a control, we performed qRT-PCR with samples of this strain in the two media. Despite presenting higher levels of the *ntrX* transcript compared to CC_WT, it was confirmed that in the engineered strain the expression of *ntrX* is also lower in the M5G medium (S1A Fig). Moreover, Western blot analysis did not detect NtrX_myc_ under phosphate-limited conditions, but it could be demonstrated that the protein is expressed in M2G (Fig 1B and 1C). Then, CC_NtrX_myc_ was grown in M5G supplemented with phosphate to match the concentration present in M2G and NtrX_myc_ was detected either after 1 h or 24 h of culture indicating that, in fact, phosphate induces NtrX_myc_ accumulation (Fig 1B).

Finally, cultures of CC_NtrX_myc_ were grown in M2G, centrifuged, and incubated in M2G or M5G. We observed that NtrX_myc_ was expressed in the M2G culture before and after resuspending the strain in the same medium, but the protein was no longer detected after 8 h in M5G (Fig 1C). When we used an initial culture in M5G, NtrX_myc_ was not expressed neither at the beginning of the assay nor after the incubation in the same medium, but it was detected after 8 h in M2G (Fig 1C). Altogether, our work points out that NtrX expression is induced under phosphate-replete conditions.

### NtrX is phosphorylated during stationary phase

**Fig 2:**
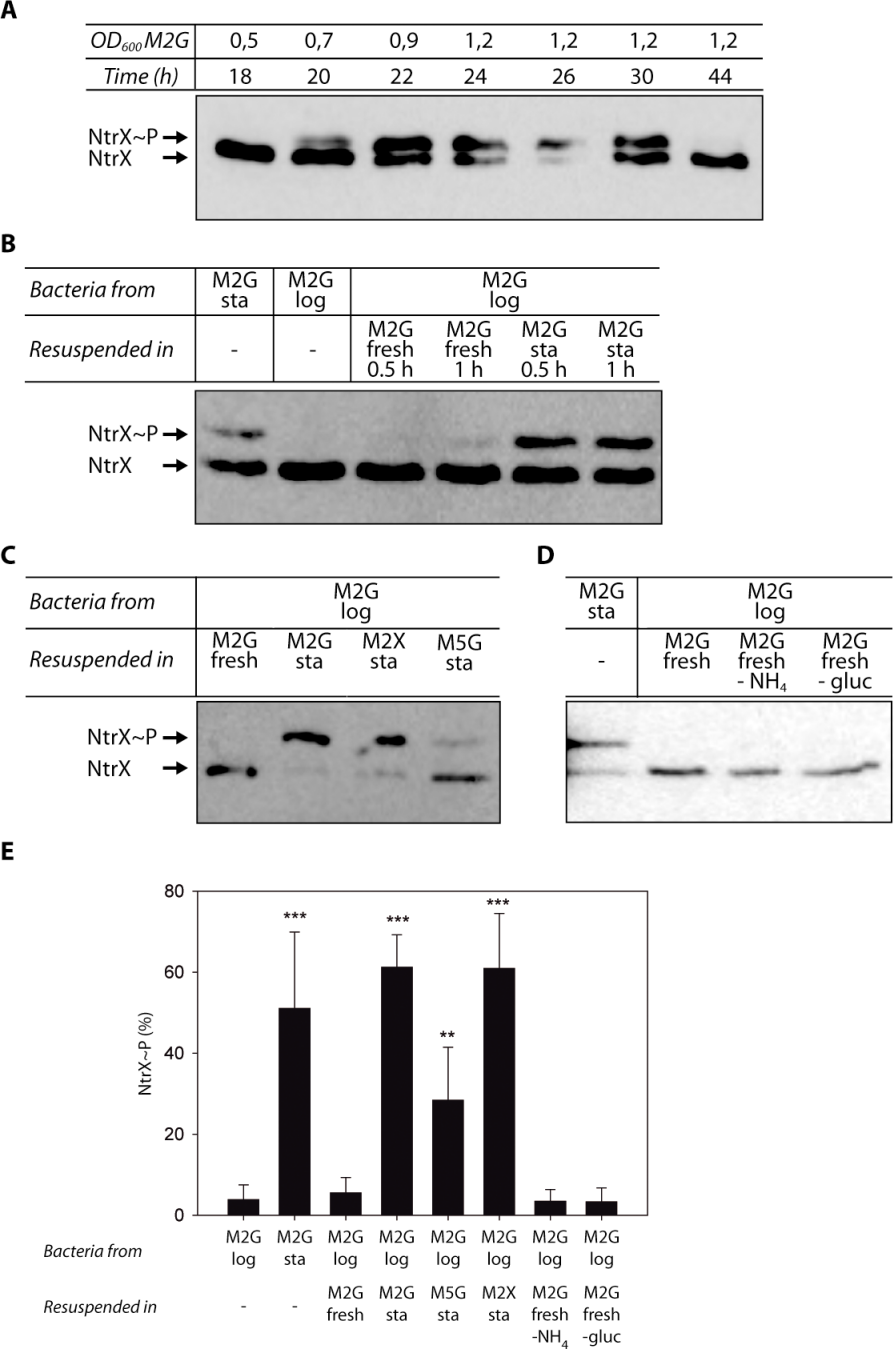
NtrX phosphorylation is achieved during the stationary phase of growth. Different samples of CC_NtrX_myc_ were analyzed by phosphoprotein affinity electrophoresis and Western blot to determine the presence of phosphorylated NtrX (the phosphorylated and non-phosphorylated forms of the protein are indicated on the left of the gels). (A) An overnight culture was diluted in fresh M2G and samples were taken at the indicated time points to determine their OD_600_ and NtrX phosphorylation. (B) M2G log-phase cultures (‘M2G log’) were centrifuged and resuspended in fresh M2G or in cell-free supernatants from cultures in stationary phase (‘M2G sta’). NtrX phosphorylation was analyzed in samples taken after 0.5 h or 1 h incubations. As controls, aliquots of the original stationary- and log-phase cultures were included. (C) Bacteria grown in M2G until logarithmic phase were centrifuged, resuspended in fresh M2G (control) or in cell-free supernatants from cultures grown until stationary phase in M2G (‘M2G sta’,), M2X (‘M2X sta’) or M5G (‘M5G sta’). Samples were obtained after an incubation period of 0.5 h. (D) Western blot of bacterial lysates obtained from cultures grown in M2G until logarithmic phase that were centrifuged and resuspended in fresh M2G, or in fresh M2G prepared without ammonium chloride (‘M2G –NH_4_’) or without glucose (‘M2G –gluc’), and incubated for 0.5 h. Each experiment from panels (A) to (D) was performed independently at least three times, and the result of one of these repetitions is shown. However, the bands of all of them were quantified and used to elaborate the histogram presented in (E). The statistical analysis was performed by a one-way ANOVA followed by a Bonferroni’s multiple comparisons post-hoc test, comparing ‘M2G sta’ to ‘M2G log’, and ‘M2G log resuspended in M2G fresh’ to all the conditions in which the ‘M2G log’ culture was resuspended. **p<0.01, ***p<0.001.

After verifying that NtrX is expressed in M2G, we studied its phosphorylation status at different stages of growth. To this end, the CC_NtrX_myc_ strain was grown in M2G and samples were taken at different times to measure their OD_600_ and to perform electrophoresis in gels with affinity for phosphoproteins. These gels were prepared with Phos-tag™, a reagent that reduces the migration of phosphorylated proteins, and NtrX_myc_ was identified by Western blot against the tag. Our experiments show that NtrX_myc_ is phosphorylated upon entry to stationary phase, it remains phosphorylated for 10 h and returns to a dephosphorylated state after a prolonged period of time (i.e. 44 h of culture) (Fig 2A). As a control, we also analyzed a stationary-phase sample of a Δ*ntrX* mutant transformed with a plasmid that codes for NtrX_myc__D53A (pMR10-NtrX_myc__D53A), in which the phosphorylatable aspartate residue was mutated for alanine. In this case, we did not observe a band of the tagged protein with reduced mobility (S2 Fig), indicating that the modification that NtrX undergoes during stationary phase is its phosphorylation, and that the Phos-tag^TM^ gels separate the phosphorylated isoform from the unphosphorylated protein.

To determine if NtrX phosphorylation was a consequence of a modification in the culture medium associated with the bacterial growth, log-phase bacteria were centrifuged and resuspended in cell-free supernatants from stationary-phase cultures. After incubating them for 0.5 h or 1 h, we observed a significant increase in NtrX_myc_ phosphorylation (Fig 2B and 2E). On the contrary, phosphorylated NtrX_myc_ (NtrX~P) was not detected after incubation of log-phase bacteria with fresh M2G medium (Fig 2B and 2E), indicating that NtrX phosphorylation is triggered by an extracellular signal that is present in the supernatants of stationary-phase M2G cultures.

Then, log-phase bacteria were resuspended in stationary-phase supernatants obtained from cultures grown in M5G, which produced a significant increase in the phosphorylation of NtrX_myc_ with respect to fresh M2G, but this increment was not as high as that observed with stationary-phase supernatants from M2G cultures (the phosphorylated fraction reached levels of 30% and 60%, respectively) (Fig 2C and 2E). The experiment was repeated by resuspending the exponential-phase bacteria grown in M2G with a supernatant obtained after growing *C*. *crescentus* until stationary phase in M2X, a minimal medium that has xylose as the carbon source. In this case, we determined a significant increase in the phosphorylated fraction of NtrX_myc_ with respect to fresh M2G, reaching a percentage of NtrX~P similar to stationary-phase M2G supernatants (Fig 2C and 2E), indicating that the signal that causes NtrX phosphorylation is produced by the bacterial metabolism using either glucose or xylose as carbon source.

In order to identify the signal, we tested conditions that are hallmarks of cultures at stationary phase. We incubated log-phase bacteria with fresh M2G prepared without glucose (M2G -gluc) or without ammonium (M2G –NH_4_) and determined that phosphorylated NtrX_myc_ was not present in any of the cell lysates obtained from these samples (Fig 2D), and that there was no significant difference with respect to resuspending the bacteria in fresh M2G (Fig 2E). These results rule out that the scarcity of glucose or ammonium might cause NtrX phosphorylation during stationary phase.

### NtrX is phosphorylated under acidic pH conditions

**Fig 3:**
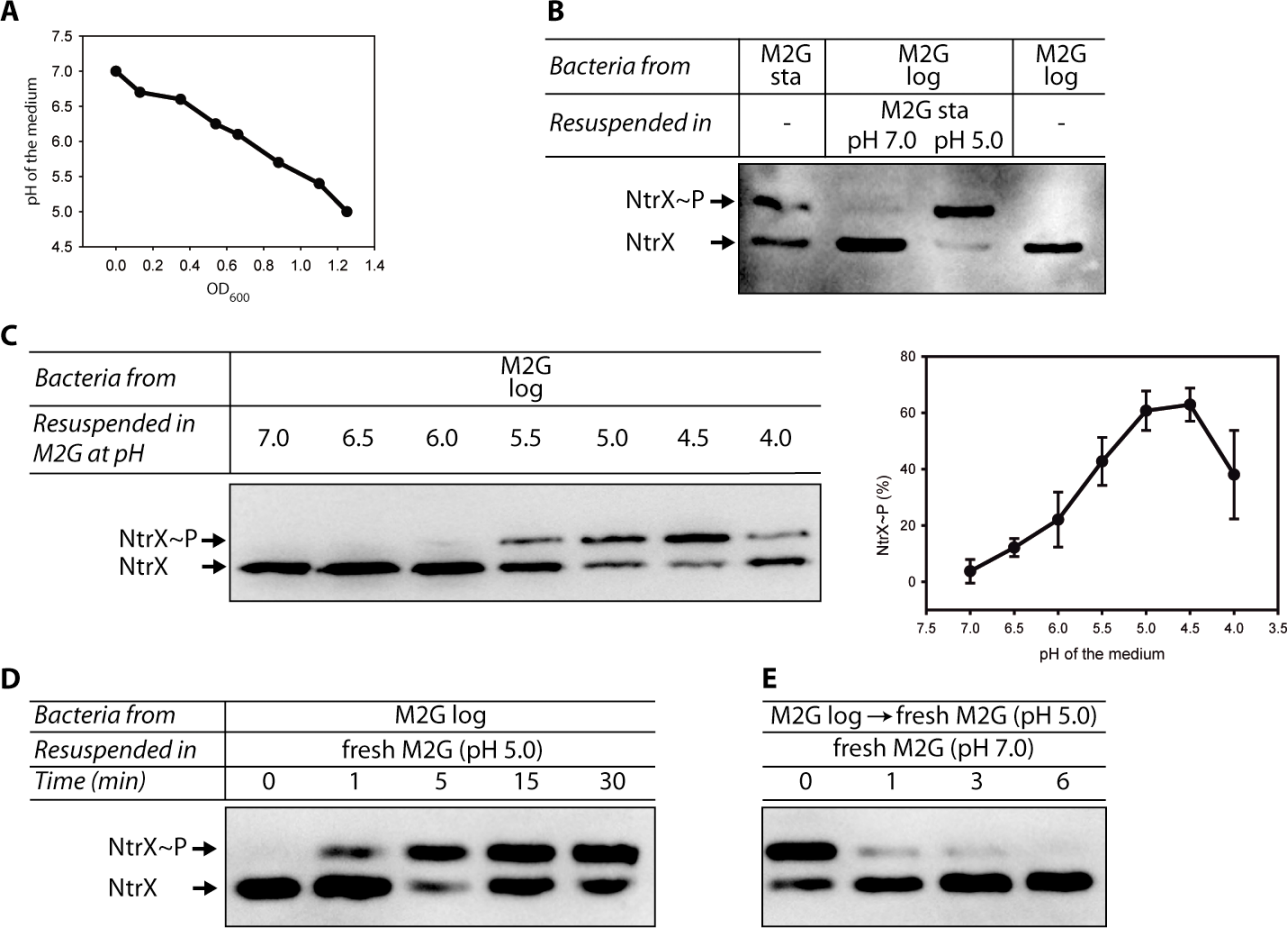
The acidification produced at stationary phase is responsible for NtrX phosphorylation. (A) Variation in the pH of the supernatant of a *C*. *crescentus* culture in M2G as a function of the bacterial optical density. (B-E) Phos-tag™ electrophoresis and Western blot of CC_NtrX_myc_ at exponential phase (‘M2G log’) treated under different conditions. (B) Bacteria were resuspended in cell-free supernatants from cultures in stationary phase (‘M2G sta’) with their pH adjusted to 7.0) or with their corresponding pH (‘pH 5.0’), and analyzed after 30 minutes. Bacteria collected from the original cultures at stationary and log phases were used as controls. (C) Exponentially growing cultures were centrifuged, resuspended in M2G with the pH adjusted to different values (indicated in the figure) and incubated for 30 minutes before Phos-tag^TM^ and Western blot (left). The intensity of the bands was quantified in several experiments and used to elaborate the plot presented on the right of the panel. (D) Kinetics of phosphorylation at pH 5.0. Log-phase CC_NtrX_myc_ was resuspended in fresh acidic M2G (pH 5.0) and aliquots were taken at different times. (E) Kinetics of dephosphorylation at pH 7.0. A log-phase culture was incubated for 30 min with fresh M2G at pH 5.0 to allow NtrX phosphorylation. Then the culture was centrifuged, resuspended in fresh M2G at pH 7.0 and incubated for the indicated periods of time when aliquots were taken to perform the Phos-tag™ electrophoresis. Each experiment was performed independently at least three times, and the results of one of these repetitions are shown.

It has been reported that glucose, as a sole organic carbon source in minimal medium, is metabolized by *C*. *crescentus* by the Entner-Doudoroff pathway and that pH decreases during culture [17]. In fact, we measured the pH of supernatants from *C*. *crescentus* cultures in M2G at different times and corroborated that the medium is acidified as the bacteria enter stationary phase (reaching a pH value of 5.0, Fig 3A). Therefore, we investigated if the exposure to acidic pH is the environmental signal that leads to NtrX phosphorylation. CC_NtrX_myc_ at exponential phase was resuspended in cell-free supernatants from cultures in stationary phase, with or without their pH adjusted to 7.0. After performing Phos-tag™ electrophoresis and Western blot analysis to these samples, we observed that NtrX_myc_ was phosphorylated only when the bacteria were incubated in the acidic supernatant (Fig 3B), confirming that the acidification produced at stationary phase is responsible for NtrX phosphorylation. To determine the pH range at which this event is triggered, log-phase CC_NtrX_myc_ was resuspended in fresh M2G with the pH adjusted to different values. The results show that NtrX_myc_ is phosphorylated under mild acidic conditions, observing the maximum phosphorylation between pH 5.0 and 4.5, which corresponds to 60% of NtrX_myc_ in the phosphorylated state (Fig 3C), as was also determined in the cultures under stationary phase (Fig 2E).

Taken into consideration that stationary-phase supernatants obtained in M5G were not efficient to phosphorylate NtrX_myc_, and that this medium is prepared with a different buffer system (Pipes instead of phosphate), we measured their pH. During early stationary phase, M5G cultures reached a pH of 6.0, a value at which the phosphorylated fraction of NtrX_myc_ is low (approximately 20% as shown in Fig 3C), explaining their poor efficiency to phosphorylate NtrX_myc_ previously (around 30% of NtrX~P, Fig 2E).

Since our experiments were performed adjusting the pH of the M2G medium with HCl, it was necessary to exclude that the chloride ions were triggering NtrX_myc_ phosphorylation. For this reason, we repeated our assays resuspending log-phase CC_NtrX_myc_ in fresh M2G that was adjusted to different pH values with acetic acid (HAc) or sulfuric acid. Regardless of the acid used, we observed maximal phosphorylation at mild acidic pH (S3 Fig), confirming that the acidic pH is the signal that causes NtrX phosphorylation and not the presence of chloride ions.

Finally, we incubated log-phase CC_NtrX_myc_ bacteria with fresh acidic M2G for different periods of time and observed that NtrX_myc_ phosphorylation takes place as soon as 1 min after the treatment (Fig 3D). Then, these treated cultures were centrifuged and resuspended in fresh M2G at pH 7.0, which caused a very fast (< 1 min) dephosphorylation of NtrX_myc_ (Fig 3E). Therefore, acidic pH is acting as a switch able to dictate the phosphorylation status of NtrX.

### *ntrX* deletion causes a decreased bacterial viability during stationary phase

**Fig 4:**
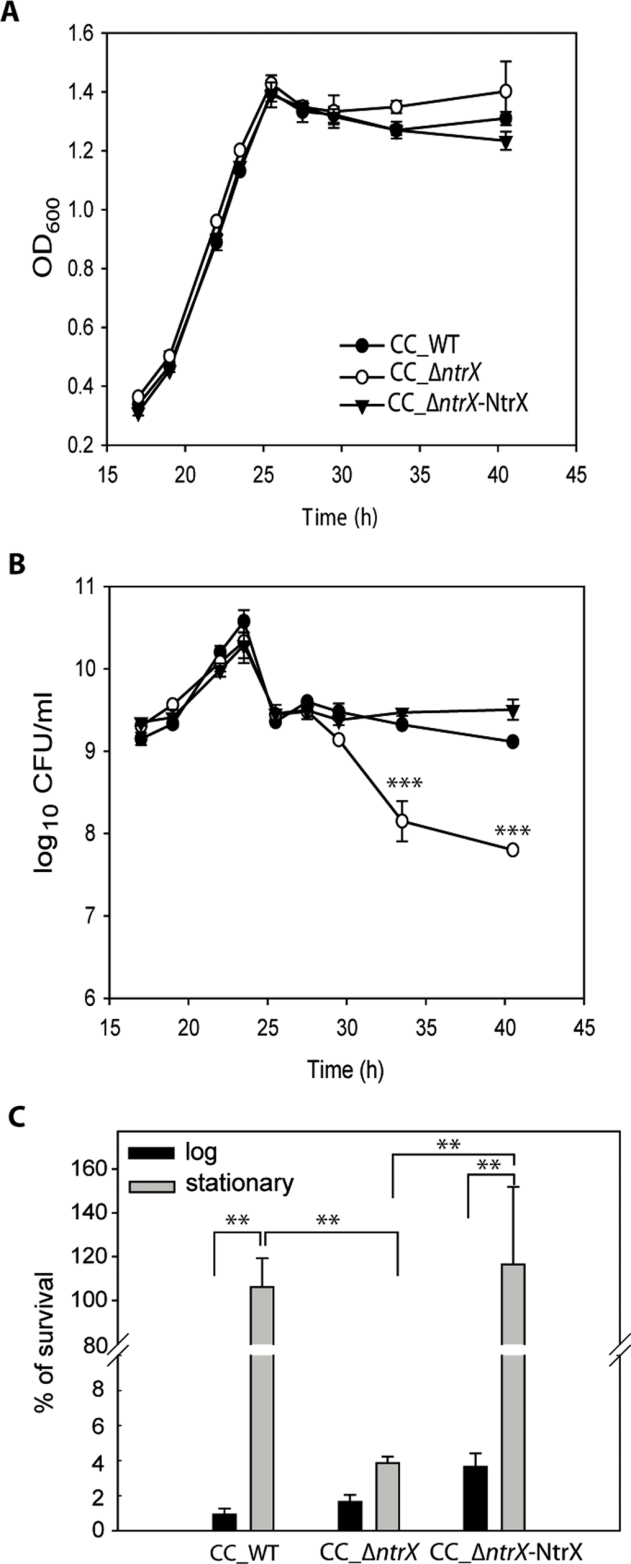
*ntrX* deletion causes a decreased bacterial viability during stationary phase and under acidic stress. Cultures of *C*. *crescentus* CB15N (WT), the Δ*ntrX* mutant strain and the complemented strain CC_ΔntrX-NtrX_myc_ (*ΔntrX*+pMR10-*ntrX*_*myc*_) were diluted to an OD_600_ of 0.005 in M2G and were incubated at 30°C with agitation. Samples were periodically taken to determine the OD_600_ (A) and bacterial viability (B) by counting colony-forming units (CFU) per ml. Each assay was performed by duplicate and the average ± SD of one representative experiment is shown. Statistical analysis was performed by a one-way ANOVA followed by a Bonferroni’s multiple comparisons post-hoc test. ***p<0.001 between CC_WT and CC_ Δ*ntrX*. (C) Bacterial survival in response to acid stress. Bacteria grown in M2G until logarithmic or stationary phases were incubated in acidic M2G (pH 4.0) for 30 minutes. At the initial and final time points the number of viable bacteria was determined and the percentage of survival was calculated. The experiment was performed by triplicate and the mean + SD of a representative experiment is shown. Data was analyzed by a two-way ANOVA followed by a Bonferroni’s multiple comparisons post-hoc test. **p<0.01.

Given that NtrX is phosphorylated during stationary phase in M2G, we wanted to establish if deleting the *ntrX* gene affects the bacterial viability at this particular culture stage. *C*. *crescentus* CB15N (CC_WT) and Δ*ntrX* (here denoted as CC_Δ*ntrX*) were grown in M2G and samples were taken at different times to measure their OD_600_ and to determine the viability by plating on solid media. The culture of CC_WT increased its OD_600_ and the number of bacteria during exponential phase, presenting a slight decrease in viability upon entry into stationary phase and a stable number of CFU at this stage during the analyzed period (Fig 4A and 4B). In contrast to previous reports that described a slower doubling time for CC_Δ*ntrX* with respect to CC_WT [4], we observed that the OD_600_ and the number of cells of the mutant strain increased during exponential phase at a rate similar to the wild type. However, during stationary phase there was a persistent and significant decrease on the amount of viable bacteria when compared to the wild-type strain (Fig 4B). Given that the OD_600_ at stationary phase is similar between CC_WT and CC_Δ*ntrX* (Fig 4A), the reduction in the number of viable cells is accompanied by an increasing amount of dead bacteria that are not lysed. Complementation of the *ntrX* deletion with the wild-type tagged gene (CC_ΔntrX-NtrX_myc_) restores the phenotype of the wild-type strain (Fig 4A and 4B). Altogether, our results show that entry into stationary phase conduces to NtrX phosphorylation and that the presence of this RR is required to sustain viability throughout this stage.

### *C*. *crescentus* NtrX is involved in acid resistance

It has been proved that *C*. *crescentus* presents an increased resistance to acid stress during stationary phase [18]. Therefore, we decided to study *ntrX* relevance in the response to a sudden exposure to acidic pH when the cells are at an exponential or stationary phases. The performed experiment consisted in growing the bacteria in M2G until they reached the desired stage, resuspending them in M2G at pH 4.0, incubating them for 30 min and determining the number of viable cells. In accordance to previous reports [18], wild-type *C*. *crescentus* presented a significant increase in its resistance to acid stress during stationary phase, while exponential cultures showed a fast death rate (Fig 4C). When CC_Δ*ntrX* was exposed to pH 4.0 for 30 min, the cultures at exponential phase presented a drastic reduction on their viability, comparable to the percentage of survival of the wild-type strain (Fig 4C). Importantly, cultures of the mutant strain at stationary phase showed a marked reduction on their viability after the acidic stress, reaching a survival percentage that is significantly lower than that corresponding to the wild-type strain under the same culture stage (Fig 4C). On the other hand, the complemented strain CC_ΔntrX-NtrX_myc_ behaves as CC_WT. This indicates that NtrX is required to elicit a response during stationary phase that leads to the increased acid resistance that characterizes this stage.

### NtrX phosphorylation is also triggered by acidic pH in *Brucella abortus*

As it has been mentioned, the NtrY/X system has been involved in numerous responses to diverse stimuli in different microorganisms. For this reason, we wanted to address if the phosphorylation of the RR by acidic pH is a singular feature of *C*. *crescentus* biology or if it is conserved in other bacteria. Our group has been studying the role of the NtrY/X system in the physiology and virulence of *B*. *abortus*, which is, as *C*. *crescentus*, an alphaproteobacteria. We reported that this pathway participates in the adaptation to low oxygen concentrations and that the intracellular PAS domain is important for this function [5]. We thought that it would be interesting to use *B*. *abortus* as another model to study the signaling triggered by acidic pH because one major mechanism of *Brucella* pathogenesis is the ability to survive in an acidic environment inside macrophages [19]. In fact, phagosome acidification is a key intracellular event to induce the expression of virulence genes [20].

**Fig 5:**
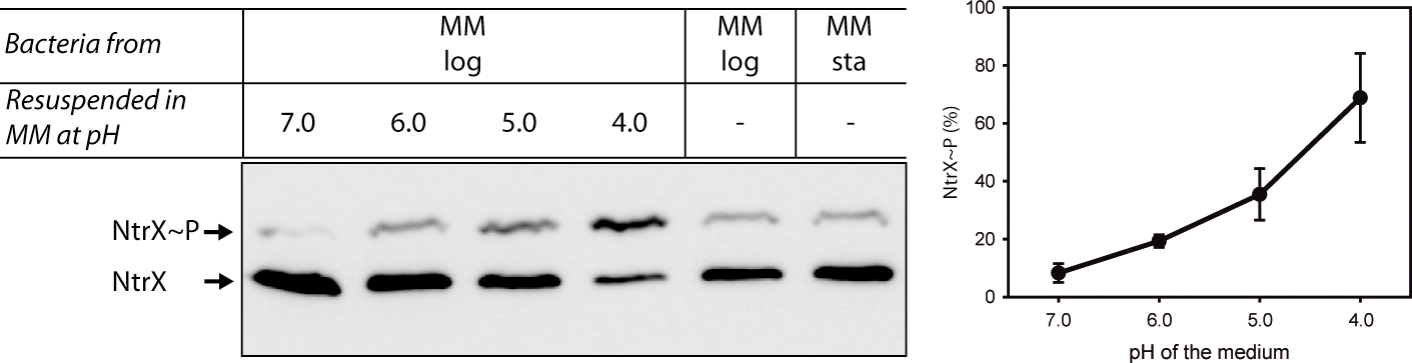
NtrX phosphorylation is also triggered by acidic pH in *Brucella abortus*. Phosphoprotein affinity gel electrophoresis followed by Western blot of samples of the *B*. *abortus* BA-NtrX_myc_ strain grown in minimal medium until logarithmic phase (‘MM log’), resuspended in fresh MM with its pH adjusted to different values and incubated for 30 min. As controls, samples of cultures at log and stationary phases were analyzed (‘MM log’ and ‘MM sta’, respectively). The phosphorylated and non-phosphorylated forms of NtrX are indicated on the left of the gels. Two independent experiments were performed and the bands were quantified to calculate mean ± SD, which were plotted in a graph presented on the right of the figure.

We constructed a *B*. *abortus* strain with a chromosomally myc-tagged NtrX protein (BA_NtrX_myc_) and grew it in minimal medium (MM). During exponential phase, the pH of the medium was close to 7.0 and it did not change significantly upon entry into stationary phase. We also analyzed samples of BA_NtrX_myc_ at different stages of the growth cycle, observing a low proportion of phosphorylated NtrX_myc_ in exponential and stationary phases (Fig 5). Then, exponential-phase cultures were centrifuged, resuspended in fresh media with the pH adjusted to different values, incubated for 30 min and used to prepare cell lysates that were subjected to Phos-tag™ electrophoresis and Western blot. The results show that NtrX_myc_ is barely phosphorylated at neutral pH (consistent with the previous results in exponential and stationary phases) and that the phosphorylated fraction increases at lower pH values, with a maximal extent achieved at pH 4.0 (Fig 5). These findings demonstrate that the triggering of NtrX phosphorylation by acidic pH observed in *C*. *crescentus* also takes place in *B*. *abortus*, pointing out to a potentially conserved role across the alphaproteobacteria class.

## Discussion

The NtrY/X TCS is an intriguing signaling pathway in bacteria as it is one of the least characterized systems. It was initially described many years ago [7], and important contributions were made recently to the general understanding of its regulation and activity [5,6,9–13,21,22]. In the present article we identify a signal that positively regulates the expression of the RR NtrX, another signal that triggers its phosphorylation, and their relevance in the bacterial physiology. It is important to highlight that our experiments provide, for the first time, direct evidence of the *in vivo* phosphorylation of NtrX.

It has been reported that NtrX expression is regulated by proline and glutamine in *E*. *chaffenssis* [10] and our group demonstrated that limited oxygen conditions induce the operon that codes for the NtrY/X TCS in *B*. *abortus* [5]. Herein, we report that the amount of NtrX in *C*. *crescentus* depends on the availability of phosphate in the medium, with high concentrations of phosphate leading to the accumulation of the RR. This finding is caused, at least in part, by an upregulation of *ntrX* transcription under phosphate-replete conditions. In this regard, it would be interesting to determine the pathway involved in this induction. For example, the PhoR/B TCS is a conserved signal transduction system that allows bacteria to respond to phosphate limitation [23], though *ntrX* has not been identified as a repressed target within the PhoB regulon [24] Therefore, the modulation of *ntrX* transcription by phosphate is not due to a direct binding of PhoB to the promoter of the NtrY/X operon, but rather through another transcriptional factor regulated by PhoB, or through a different signal transduction pathway.

On the other hand, even though we determined that the level of the *ntrX* transcript in M5G is approximately 30% of that corresponding to M2G, the protein could not be detected by Western blot under the same phosphate-limited conditions. Besides, the level of the NtrX_myc_ protein became undetectable when CC_NtrX_myc_ was grown in M2G and then incubated in M5G for 8 h. However, when CC_NtrX_myc_ was grown in M5G and then incubated in M2G for the same period of time, the amount of NtrX_myc_ was not restored to the levels of the protein in M2G at time zero. All these observations might indicate that the concentration of phosphate could also modulate the proteolysis rate of NtrX.

One of the most important findings that we present in this article is the triggering of NtrX phosphorylation when *C*. *crescentus* is under acidic conditions, a typical feature of its growth during stationary phase in M2G medium. It remains to be determined if periplasmic protons *per se* are the signal involved, but some of our results support this notion. The fact that fresh acidic M2G medium, which contains exclusively glucose and salts and was adjusted with HCl (not an organic acid), is enough to lead to the phosphorylation of NtrX indicates that the signal sensed is not an organic molecule produced and secreted by the bacteria during stationary phase. In fact, the same effect was obtained when the pH of the medium was adjusted with H_2_SO_4_ or HAc. On the other hand, the fact that neutralophilic bacteria generally maintain their cytoplasmic pH values in a narrow range despite the external pH [25] indicates that it is likely that the periplasmic pH is the environmental cue relevant to NtrX phosphorylation rather than the cytoplasmic pH. Nevertheless, the cytoplasm of some bacteria, such as *Salmonella enterica*, is acidified upon acid stress [26], but this phenomenon requires several minutes to occur (approximately 120 minutes to decrease the pH in 0.75 units [26]) in contrast to the fast phosphorylation of NtrX that takes place as soon as 1 min after incubation in an acidic medium. Of note, we showed that NtrX is rapidly dephosphorylated upon acidification and reincubation in a neutral-pH medium, indicating that NtrX~P is being the substrate of a phosphatase.

Despite our efforts to obtain a *C*. *crescentus* strain with the *ntrY* gene deleted and coding for a myc-tagged NtrX, we could not generate this mutant to confirm that NtrY is the sensor kinase that detects acidic pH and phosphorylates NtrX as a consequence. Other histidine kinases have been reported to respond to low pH such as PhoQ [27], PmrB [28], ArsS [29] and EvgS [30]. All of them contain periplasmic domains that allow the detection of an acidic environment (although it has been recently proposed that the activation of PhoQ occurs in response to a reduction in the cytoplasmic pH [31]). NtrY has a periplasmic domain with a secondary structure prediction [32] that classifies it within the PDC family [33], which groups extracellular sensor domains from PhoQ, DcuS and CitA. Taking into consideration that NtrY is a histidine kinase involved in redox sensing through its intracellular PAS domain [5], the potential role of its periplasmic domain in detecting acidic pH would imply that NtrY is able to integrate different environmental signals.

Overall, our results lead us to postulate that *C*. *crescentus* NtrX orchestrates an adaptive response to acidic pH that initially requires the phosphorylation of the RR but is sustained over the time without NtrX~P, given that the protein is phosphorylated upon entry to stationary phase and it becomes dephosphorylated after several hours. This initial response would allow the bacteria to survive for a prolonged period under stationary phase, since deleting *ntrX* produces a decreased viability after 5 h at this phase. Also, this response would be responsible for the acquisition of the characteristic acid-stress resistance observed in *C*. *crescentus* at stationary phase [18], given that the mutant strain CC_Δ*ntrX* does not present this phenotype. We did not observe differences between the mutant and wild-type strains when the experiment of acidic resistance was performed with bacteria at log phase, possibly because the stress is very drastic and the bacteria at this stage are too susceptible and die before the adaptive mechanisms are activated.

In spite of our progress, it still remains to elucidate which are the molecular mechanisms involved in the response to acidic pH in *C*. *crescentus* and the role that NtrX has in their regulation. It has been proposed that glutamate, arginine and lysine decarboxylases contribute to pH homeostasis in *E*. *coli* [34], but these enzymes are not encoded in *C*. *crescentus* genome. Also, under conditions of acid challenge *E*. *coli* increases the expression of other cytoplasmic enzymes that catalyze reactions that consume protons and of respiratory chain complexes that pump protons out of the cell [25]. It is possible that the role of NtrX in the adaptation to acidic pH is linked to those strategies, since we have described that the NtrY/X TCS of *B*. *abortus* activates the expression of denitrification enzymes (which catalyze reactions that require protons) [5] and of the ccoN cytochrome oxidase (that pumps protons out) [6].

Our approach to study *C*. *crescentus* using defined minimal media proved to be very useful to dissect different components that promote NtrX expression and phosphorylation. However, *ntrX* is essential for growth in rich media (PYE) [3], where we determined that the pH is neutral and no acidification is produced during bacterial growth. Therefore, unphosphorylated NtrX must have a key role in the bacterial physiology that still has to be discovered.

Another highlight of our work is that we demonstrate that acidic pH is also capable of triggering NtrX phosphorylation in the pathogenic bacterium *B*. *abortus*. Since *C*. *crescentus* and *B*. *abortus* belong to the same class but are not closely related (rhizobial and caulobacteral orders, respectively), our results could indicate that the phosphorylation of NtrX upon acidification, and its role in the adaptation to low pH, are conserved across the alphaproteobacteria class. Given that low pH acts as an intracellular signal for the expression of genes involved in survival and multiplication of *B*. *abortus* within the phagocytic cell [35], it would be interesting to determine if NtrX is involved in the regulation of this virulence-related transcriptional network. Also, some mechanisms have been proposed to protect *Brucella* against the adverse effects of acidification (such as the expression of urease) [36] and it would be important to prove if NtrX is required for their induction.

In conclusion, we have contributed to deepen the knowledge on the NtrY/X pathway by identifying the phosphate concentration as a signal that is necessary for the expression of NtrX in *C*. *crescentus*, and acidic pH as a trigger of NtrX phosphorylation in two different species of alphaproteobacteria. We also demonstrate that NtrX has an important role in the adaptation to environments with low pH. It is noteworthy that we used a direct approach to detect NtrX~P, which led us to postulate that the environmental pH acts as a switch capable of regulating the phosphorylation status of NtrX. Therefore, we have outlined an experimental set-up with the RR in two defined states (unphosphorylated in M2G at pH 7.0, and phosphorylated in M2G at pH 5.0) that will be valuable to engage the elucidation of the poorly-characterized NtrX regulon.

## Supporting information

S1 Fig Levels of the *ntrX* transcript in the engineered strain CC_NtrX_myc_ and loading controls of Fig 1. (A) The strains CC_WT and CC_NtrX_myc_ were grown until stationary phase in M2G and M5G. Total RNA was extracted and the levels of the *ntrX* transcript were determined in both strains and media by qRT-PCR. The data represent the mean ± standard deviation of an experiment performed in triplicate. (B and C) The same volumes of the samples analyzed in Fig 1B and 1C (Results) were loaded in SDS-PAGE gels that were stained with Coomassie Brilliant Blue. MWM: molecular weight marker.
(TIF)

S2 Fig Phos-tag^TM^ gels separate phosphorylated NtrX. A *C*. *crescentus* Δ*ntrX* mutant strain that had been transformed with the plasmid pMR10 coding for NtrX_myc__D53A (CC_ Δ*ntrX*-NtrX_myc__D53A) and the strain CC_NtrX_myc_ were grown until stationary phase in M2G, and samples were subjected to Phos-tag^TM^ electrophoresis and Western blot analysis.
(TIF)

S3 Fig NtrX is phosphorylated under acidic pH regardless of the acid used to adjust the pH of the medium. CC_NtrX_myc_ was grown in M2G until logarithmic phase and it was resuspended in fresh M2G with the pH adjusted to different values (indicated in the figure) with acetic acid (HAc, upper panel) or sulfuric acid (lower panel). After a 30 min incubation aliquots were removed and analyzed by Phos-tag^TM^ gels and Western blot.
(TIF)

## Abbreviations

HK: histidine kinase
NtrX~P: phosphorylated NtrX
OD_600_: optical density at 600 nm
RR: response regulator
TCS: two-component system
