# Supplementary figures and images for: Acidic pH triggers the phosphorylation of the response regulator NtrX in alphaproteobacteria
Source: PLoS One. 2018 Apr 10;13(4):e0194486. doi: 10.1371/journal.pone.0194486 (PMC5892882; doi:10.1371/journal.pone.0194486)

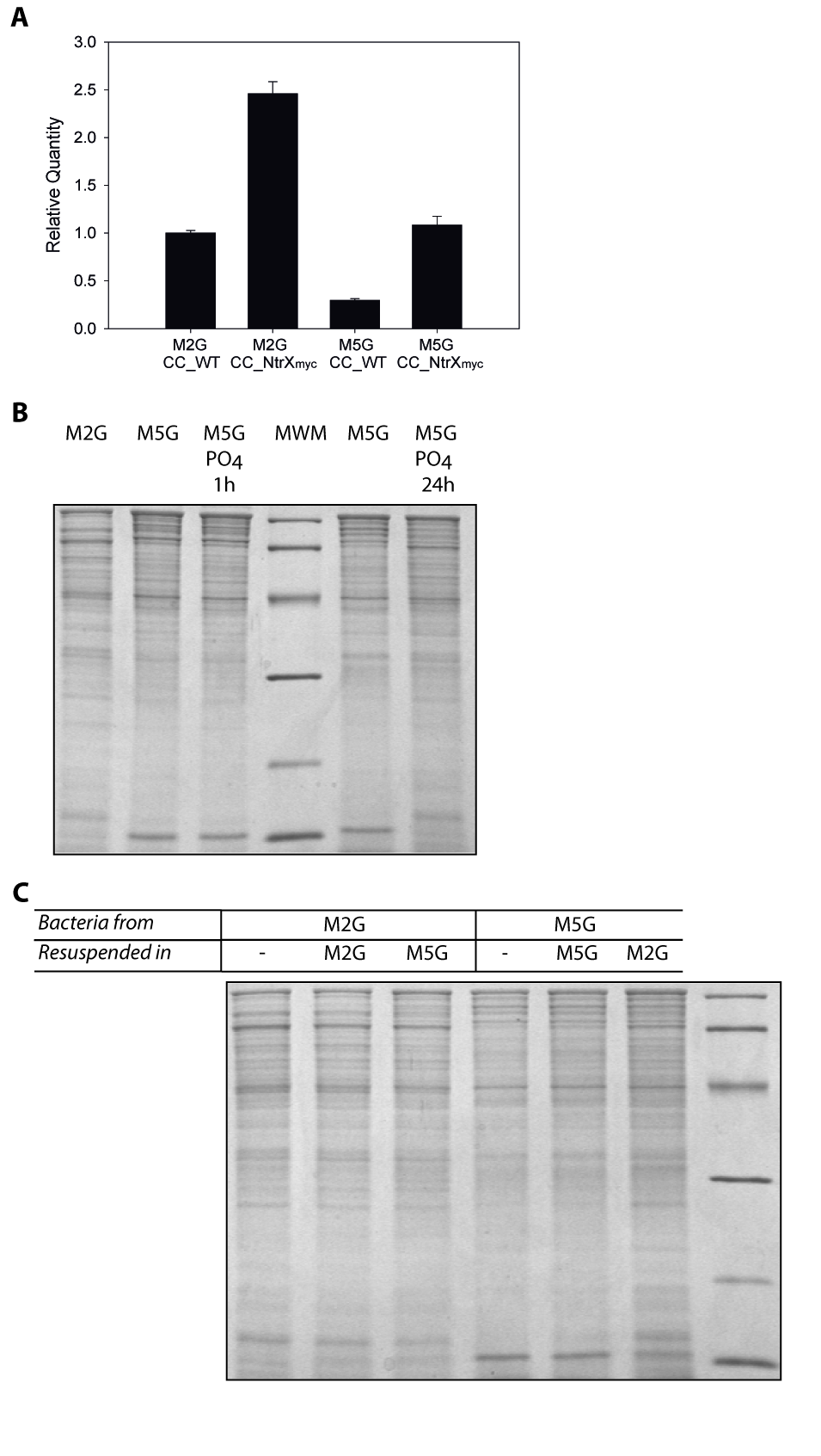

Supplement: S1 Fig — (A) The strains CC_WT and CC_NtrXmyc were grown until stationary phase in M2G and M5G. Total RNA was extracted and the levels of the ntrX transcript were determined in both strains and media by qRT-PCR. The data represent the mean ± standard deviation of an experiment performed in triplicate. (B and C) The same volumes of the samples analyzed in Fig 1B and 1C (Results) were loaded in SDS-PAGE gels that were stained with Coomassie Brilliant Blue. MWM: molecular weight marker. (TIF) [file pone.0194486.s001.tif]

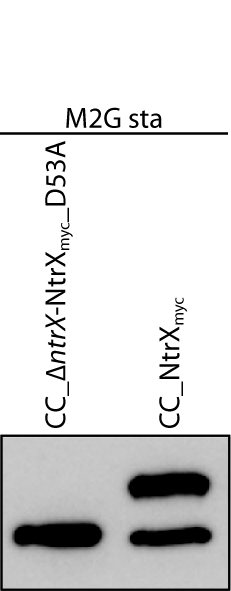

Supplement: S2 Fig — A C. crescentus ΔntrX mutant strain that had been transformed with the plasmid pMR10 coding for NtrXmyc_D53A (CC_ ΔntrX-NtrXmyc_D53A) and the strain CC_NtrXmyc were grown until stationary phase in M2G, and samples were subjected to Phos-tagTM electrophoresis and Western blot analysis. (TIF) [file pone.0194486.s002.tif]

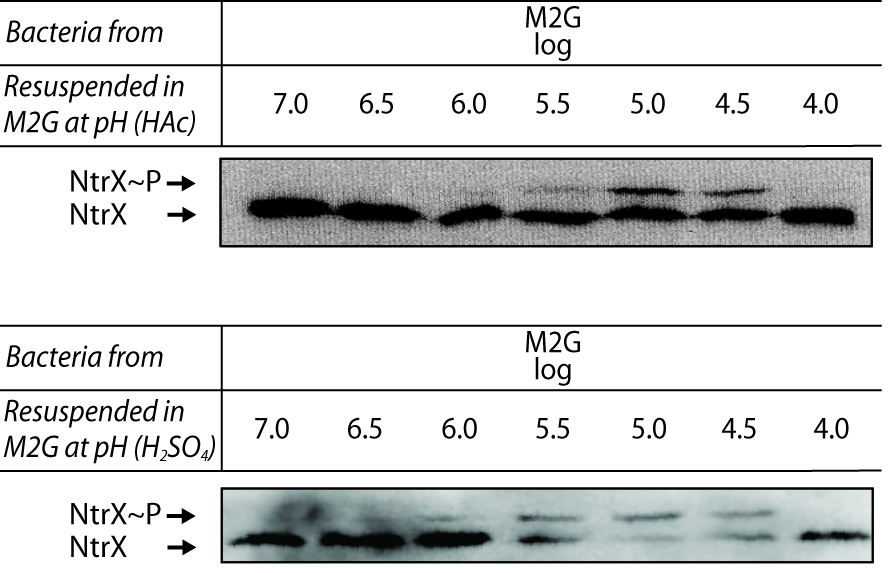

Supplement: S3 Fig — CC_NtrXmyc was grown in M2G until logarithmic phase and it was resuspended in fresh M2G with the pH adjusted to different values (indicated in the figure) with acetic acid (HAc, upper panel) or sulfuric acid (lower panel). After a 30 min incubation aliquots were removed and analyzed by Phos-tagTM gels and Western blot. (TIF) [file pone.0194486.s003.tif]
